# Comparing the Diagnostic Value of Serum D-Dimer to CRP and IL-6 in the Diagnosis of Chronic Prosthetic Joint Infection

**DOI:** 10.3390/jcm9092917

**Published:** 2020-09-10

**Authors:** Thomas Ackmann, Burkhard Möllenbeck, Georg Gosheger, Jan Schwarze, Tom Schmidt-Braekling, Kristian Nikolaus Schneider, Adrien Frommer, Ralf Dieckmann, Christoph Theil

**Affiliations:** 1 Department of General Orthopedics and Tumor Orthopedics, Muenster University Hospital, 48149 Muenster, Germany; burkhard.moellenbeck@ukmuenster.de (B.M.); georg.gosheger@ukmuenster.de (G.G.); jan.schwarze@ukmuenster.de (J.S.); tom.schmidt-braekling@ukmuenster.de (T.S.-B.); Kristian.Schneider@ukmuenster.de (K.N.S.); adrien.frommer@ukmuenster.de (A.F.); r.dieckmann@bk-trier.de (R.D.); Christoph.Theil@ukmuenster.de (C.T.); 2 Department of Orthopedic Surgery, Barmherzige Brüder Hospital, 54292 Trier, Germany

**Keywords:** periprosthetic joint infection, CRP, D-dimer, interleukin-6, revision joint arthroplasty

## Abstract

**Introduction:** D-dimer is a diagnostic criterion for periprosthetic joint infection (PJI) of the Musculoskeletal Infection Society (MSIS) in 2018. The aim of this study was to evaluate the serum D-dimer values in comparison to C-reactive protein (CRP) and interleukin-6 (IL-6) for the diagnosis of PJI. **Materials and Methods:** We included 119 patients (50 women, 69 men; 71 knees, 48 hips) undergoing revision arthroplasty with preoperative assessment of CRP, IL-6, and serum D-dimer. Cases were classified as infected or aseptic based on the MSIS criteria of 2018. Receiver operating curves and Youden’s index were used to define an ideal cut-off value and sensitivity and specificity for the individual parameters, and respective combinations were calculated using cross-tables. **Results:** The median D-dimer level (2320 vs. 1105 ng/mL; *p* < 0.001), the median CRP level (4.0 vs. 0.5 mg/dL; *p* < 0.001), and the median IL-6 level (21.0 vs. 5.0 pg/mL; *p* < 0.001) were significantly higher in the group of PJI compared to the group with aseptic failure. The calculated optimal cut-off values were 2750 ng/mL (AUC 0.767) for D-dimer, 1.2 mg/dL (AUC 0.914) for CRP, and 10.0 pg/mL (AUC 0.849) for IL-6. D-dimer showed a sensitivity of 38% and specificity of 94%, whereas the CRP and IL-6 had sensitivities of 88% and 76%, and specificities of 87% and 92%, respectively. **Conclusion:** In comparison with CRP and IL-6, serum D-dimer showed low sensitivity and specificity in our cohort. While CRP and IL-6 combination had the highest sensitivity, a combination of Il-6 and D-dimer or CRP and IL-6 had the highest specificity.

## 1. Introduction:

Periprosthetic joint infection (PJI) is a serious complication that can occur after total hip arthroplasty (THA) or total knee arthroplasty (TKA). The number of patients undergoing revision surgeries for PJI is expected to rise over the coming years, and PJI already is one of the main causes of revision surgeries today [1,2]. The treatment of PJI can have extremely negative effects on the emotional, social, physical, and economic aspects of patients’ lives [3].

The preoperative diagnosis of PJI is crucial for adequate treatment, but it is often challenging because there is no optimal test available. The correct diagnosis of PJI is based on a combination of clinical findings as well as serum, synovial, and microbiological tests [4,5,6]. While synovial and microbiological testing is the most reliable method, it is invasive and there is the risk of “dry taps” in joint aspirations and culture-negative infections necessitating the use of a combination of serum parameters to establish the diagnosis. Most commonly, the diagnosis is established using the criteria published by the Musculoskeletal Infection Society (MSIS) and the International Consensus Meeting (ICM) proceedings [7,8]. One novel parameter that has been included in these criteria is the serum D-dimer, which has been identified as a marker of systemic inflammation [9], while traditionally this fibrin degradation product indicates fibrinolysis. Shahi et al. [10] investigated the use of D-dimer in the diagnosis of PJI and found a superior sensitivity (89%) and specificity (93%) outperforming C-reactive protein (CRP) and erythrocyte sedimentation rate (ESR), which are usually tested in the serum. However, the role of D-dimer, particularly regarding its sensitivity and specificity, has been discussed controversially in the literature [10,11,12], with poorer diagnostic value being reported. Additionally, the ideal combinations of serum parameters, including D-dimer, for the diagnosis of PJI remain unknown [13], whereas the diagnostic value of CRP and interleukin-6 (IL-6) has already been proven in numerous studies [7,8,14,15]. The meta-analysis of Yoon et al. showed a good pooled sensitivity of 83% and specificity of 91% for IL-6 in the diagnosis of PJI.

The aim of this study is to investigate the performance of plasma D-dimer in the diagnosis of PJI in comparison with serum CRP and IL-6, to identify an optimal diagnostic cut-off value, and to evaluate an ideal combination of the serum markers.

## 2. Materials and Methods

### 2.1. Study Design

This retrospective investigation of a prospectively maintained local arthroplasty database was approved by the institutional review board of the authors’ institution (local ethical committee ref. no. 2019-666-f-S), and the study was registered in the German Clinical Trials Register (registration number: DRKS00021038). A specific funding was not received for this study. The study was conducted according to the principles of the World Medical Association Declaration of Helsinki, and written consent was obtained from all the participants.

All patients with a potential indication for prosthetic revision surgery either for chronic PJI with a minimum of three months of symptoms or fistulating infection in comparison with aseptic causes routinely undergo tests of serum and synovial parameters, as well as microbiologic culturing prior to revision surgery in order to confirm or rule out infection. At the authors’ institution, serum CRP and serum IL-6 are determined and all patients undergo preoperative joint aspiration. Synovial fluid leukocyte count and percentage of neutrophiles are measured and the synovial fluid is cultured for 14 days on Columbia agar, chocolate agar, and Schaedler agar. Serum CRP was measured by latex-enhanced immunoturbidimetric assays on the cobas^®^ c 702 chemistry analyzer (Roche Diagnostics GmbH, F. Hoffmann–La Roche, Ltd., Mannheim, Germany) and IL-6 by electrochemiluminescence immunoassays on the cobas^®^ e 801 chemistry analyzer (Roche Diagnostics GmbH, F. Hoffmann–La Roche, Ltd., Mannheim, Germany). Based on these parameters using the MSIS criteria of 2018, the respective arthroplasties were considered infected or non-infected prior to surgery. Patients with chronic systemic inflammation such as rheumatoid arthritis, those who had surgery within the last 3 weeks, those with malignancies, those with inflammatory diseases of other organs such as pneumonia and urinary tract infection, and those with a history of hypercoagulation disorders were excluded from the study. The other patients that were planned for revision arthroplasty in our clinic were included. All patients that had a confirmed chronic PJI underwent a two-stage exchange with removal of all foreign material and implantation of a spacer, while all patients with aseptic prosthetic failure underwent single-stage revision surgery.

Based on the recommendations of the MSIS criteria and the recommendations from the ICM, the authors added a D-dimer test to the algorithm of this study. D-dimer in citrate plasma was determined by the STA^®^-Liatest^®^ D-Di Plus (fibrinogen equivalent units), as well as by an immunoturbidimetric assay, on the STA R Max^®^ hemostasis analyzer (Stago, Île-de-France, France). For the final classification of a prosthetic failure as PJI or aseptic failure, intraoperative clinical findings with a minimum of five intraoperative tissue samples for microbiology cultures were taken, and another tissue sample was taken for histological purposes, again applying the criteria by the MSIS.

The authors recorded the patients’ demographic details in an electronic database.

### 2.2. Statistical Analysis

Data collection and statistical analysis were performed using Excel (Microsoft Corporation, Redmont, Washington, DC, USA) and Statistical Package for the Social Sciences Statistics for Windows version 25 (IBM Corporation, Armonk, NY, USA). All patient records were anonymized prior to analysis. Descriptive statistics and the Shapiro–Wilk test were used to analyze distribution of data. The means and ranges were calculated for parametric data; the medians and 25–75% interquartile ranges (IQRs) were obtained for non-parametric data. The non-parametric analyses were performed using the Mann–Whitney U test. Frequencies were given for categorical variables that were compared in contingency tables using the chi-squared test.

Statistical evaluation was performed with receiver operating curve (ROC) analyses with presentation of the area under the curve (AUC). The Youden’s index was used to determine the optimal cut-off value for D-dimer, CRP, and IL-6. Based on the cut-off values, the sensitivity, specificity, positive predictive value (PPV), and negative predictive value (NPV) of the serum markers were calculated from contingency tables. In addition, the authors determined the sensitivity, specificity, PPV, and NPV of D-dimer using the cut-off value proposed in the MSIS criteria 2018 (>860 ng/mL)^7^ and the potential optimal combinations among the serum markers. Statistical significance was defined as *p* ≤ 0.05. While all patients had D-dimers and CRP assessed prior to surgery, there were 8 missing values for interleukin-6.

## 3. Results

### 3.1. Demographic Details

In total 119 patients (71 TKA and 48 THA) were included in this study. Fifty-two patients (30 TKA and 22 THA) were assigned to the PJI group, while the remaining 67 patients (41 TKA and 26 THA) were assigned to the non-PJI group. The median age was 70.5 years (IQR, 60.5–83.7 years) for the PJI group and 68.0 years (IQR, 61.0–76.0 years) for the non-PJI group. Among the patients in the PJI group, 30 were males and 22 were females with a median BMI of 30.9 kg/m^2^ (IQR, 27.0–38.4 kg/m^2^). Thirty-nine males and 28 females with a median BMI of 28.4 kg/m^2^ (IQR, 25.5–31.9 kg/m^2^) were in the non-PJI group. There was a significant difference for the BMI (*p* = 0.008) between both groups. In terms of age, sex, prostheses, and the affected joint, no statistical differences between the PJI and non-PJI groups were found (Table 1).

### 3.2. Preoperative Serum Parameters

The median plasma D-dimer level was significantly higher (*p* < 0.001) in the patients diagnosed with PJI (2320 ng/mL (IQR, 1410–3350 ng/mL)) compared to the patients with aseptic failure (1105 ng/mL (IQR, 678–3350 ng/mL) (*p* < 0.001). Furthermore, the median concentrations of CRP (4.0 mg/dL (IQR, 1.8–8.7 mg/dL) vs. 0.5 mg/dL (IQR, 0.5–0.825 mg/dL), *p* < 0.001) and IL-6 (21.0 pg/mL (IQR, 9.00–37.00 pg/mL) vs. IL-6 (5.0 pg/mL (IQR, 2.8–7.0 pg/mL), *p* < 0.001) were significantly higher in patients with PJI compared to patients with aseptic revision.

**Figure 1:**
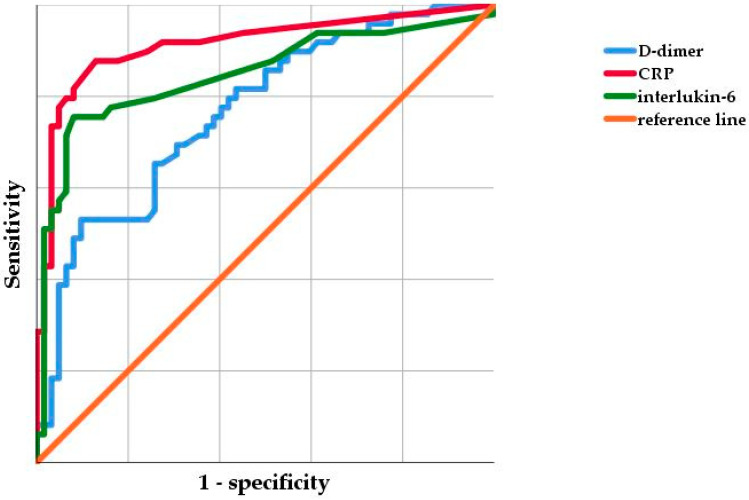
The receiver operating curves of D-dimer, C-reactive protein, and interleukin-6. CRP, C-reactive protein. The scale is in 0.2 increments from 0–1.

The ROC curve analysis (Figure 1) revealed the lowest AUC for serum D-dimer, at 0.767 (95% confidence interval (95% CI), 0.680–0.855). The highest AUC was shown for serum CRP with 0.914 (95% CI, 0.855–0.973), followed by IL-6 with 0.849 (95% CI, 0.769–0.928). Using Youden’s index, the optimal cut-off values were 2750 ng/mL, 1.2 mg/dL, and 10.0 pg/mL, for D-dimer, CRP, and IL-6, respectively, discriminating between PJI and aseptic failure.

Using the calculated cut-off values, the sensitivity and specificity for each parameter were calculated (Table 2). D-dimer had a sensitivity of 38% and specificity of 94% to detect PJI. The sensitivity and specificity of CRP were 88% and 87% and 76% and 92% for IL-6, respectively. When evaluating potential combinations of serum markers, we found that the combination of CRP and IL-6 showed the highest sensitivity (74%), followed by the combined diagnostic value of CRP and D-dimer (35%) and lastly by IL-6 in combination with D-dimer (33%). The combination of IL-6 and D-dimer (97%) showed the highest specificity in diagnosing PJI equal to CRP with IL-6 (97%). The specificity of the combination of CRP and D-dimer was marginally smaller with 96%.

Considering that the cut-off value (2750 ng/mL) from the present cohort was much higher than the value (860 ng/mL) recommended by the MSIS, the authors decided to evaluate the recommended cut-off value of the MSIS with the present cohort as well. This resulted in a high sensitivity of 92%, but poor specificity of 39% in diagnosing PJI.

## 4. Discussion

Accurately diagnosing PJI is a challenge and the search for potential markers is still ongoing [13,16,17,18,19]. D-dimer was included in the diagnostic criteria for PJI proposed by the MSIS and in the ICM criteria considering its excellent reported sensitivity and specificity [7,10]. Contrary to these findings, the authors found a much higher calculated cut-off value (2750 ng/mL) resulting in a very low sensitivity of 38%; whereas the specificity was very good with 94%. However, even if the cut-off value proposed by the MSIS was applied, only the sensitivity improved, while consequently, the specificity decreased. Furthermore, it was shown that the combination of CRP and IL-6 achieved the best results in the diagnosing PJI with a sensitivity of 75% and specificity of 97%.

Serum D-dimer has been investigated in previous studies following its inclusion in the MSIS diagnostic criteria for PJI in 2018 [11,12,16,17,18]. In the literature, there are differing opinions about its optimal cut-off value and its sensitivity and specificity (Table 3). In two previous studies [16,17], D-dimer demonstrated high sensitivities (87.50% and 92.72%) and specificities (89.19% and 74.63%) in diagnosing PJI; whereas other studies [11,12,18] revealed that D-dimer demonstrated poor accuracy in discriminating septic cases from aseptic cases.

It currently seems to be unclear what the optimal cut-off value for D-dimer is and what additional use in comparison with other serum markers the inclusion of D-dimers has. One potential reason for this is that as a marker of systemic fibrinolysis, it possibly can be easily influenced by patients’ age, sex, and ethnicity [19,20]. Four of the studies mentioned investigated D-dimer in an Asian population and one recruited their patients from an American population, which may have been predominantly Caucasian and African–American. However, it is well known that the level of D-dimer can vary in different ethnic groups [20]. As this is the first study to analyze a European, specifically German, population, it is possible that the D-dimer levels are higher in this patient group, although we must acknowledge the hypothetical nature of this notion and that usually cut-off values derived from Caucasian populations are used without adaptation for European patients; therefore, the influence of genetic or ethnic variability in D-dimers may well be quite limited. However, future studies should further investigate whether D-dimer in patients treated for PJI may be higher in European patients.

We did not find any differences in D-dimers levels based on sex or age which is in concordance with previous studies, although it is known that females and older patients can have higher serum levels of D-dimer when used in the diagnosis of thrombosis or embolism [19,21]. Therefore, in internal medicine, age-adjusted D-dimer concentrations are used to diagnose pulmonary embolism or deep vein thrombosis resulting in much higher cut-off values for older patients [21]. This should also be taken into account when using D-dimer for PJI, particularly considering that the median patient age in this study was over 70 years at the time of surgery, and future studies are necessary to investigate the serum level of D-dimer for different age groups and whether an age-adjusted cut-off might be more appropriate in the diagnosis of PJI.

When evaluating the D-dimer levels, surgeons must consider other possible influencing factors such as patient obesity, use of anticoagulants, and the role of different infecting organisms [16,22,23]. While it is known that patients with obesity have a 2–4-fold risk for PJI [24,25], obesity might also affect D-dimer levels which questions the use of D-dimer in this patient subgroup. Therefore, obese patients were excluded in a previous study, resulting in a lower mean BMI that might be unrealistic for patients undergoing surgery for PJI at least in Europe or North America [26]. Considering that the BMI of patients with PJI was 2.5kg/m^2^ higher compared to those undergoing aseptic revision, it may represent a confounding factor resulting in a higher level of D-dimers. While this aspect limits the generalizability of the cut-off value found in the present cohort, we believe that treating obese patients rather represents the everyday practice of a revision arthroplasty center [27,28], and the utility of a diagnostic test that excludes patients with grade I obesity is questionable. However, the D-dimer might still be useful in obese patients, but an adjusted higher cut-off value resulting in a lower sensitivity as found in the present study should be evaluated.

Besides a higher BMI, cardiovascular comorbidities requiring anticoagulation are quite common in patients undergoing revision arthroplasty [29,30]. Different widely used anticoagulants such as warfarin and other vitamin K antagonists or factor X inhibitors can inhibit the activation of the coagulation and fibrinolytic pathways, resulting in decreased D-dimer levels [31]. Additionally, it is possible that some comorbid conditions have an effect on D-dimer production themselves, such as autoimmune disease or history of malignant tumors [11]. As comorbid conditions and the use of anticoagulation is frequent in elderly patients undergoing revision arthroplasty, the definition of a precise, fixed cut-off value of D-dimer as a diagnostic tool for PJI appears difficult. However, as in previous studies, these patients were not included for the assessment of D-dimer; nonetheless, surgeons should be aware of this limitation when using D-dimer. Furthermore, due to the limited patient number that was available for infections caused by specific organisms, we did not analyze the impact of highly virulent or low-grade organisms or atypical organisms on D-dimer. It is possibly though that for some infections D-dimer might have an improved diagnostic accuracy compared to other serum parameters such as CRP, which has been shown to be unaltered in some low-grade infections [32].

The diagnostic algorithm used at our department relies on CRP and IL-6 testing that was found to be the most specific combination although sensitivity remains far from perfect at around 75%. Previous studies [32,33,34] have highlighted that low-grade infections might not result in elevated CRP or IL-6; however, due to limited numbers these findings should be evaluated for specific organism in future studies. It remains of paramount importance to use a combination of serum markers, synovial parameters, and microbiology as recommend by the MSIS and ICM criteria. Despite all efforts in preoperative diagnosis, some patients might require open biopsy [4] or additional testing such as next generation sequencing [5] or alpha defensin [35] that were not routinely used in our algorithm. Furthermore, it can be more useful in chronic, non-fistulating infection to perform repeat joint aspiration in order to obtain a synovial fluid sample considering the excellent diagnostic value [36]. On the other hand, CRP and IL-6 combination displayed excellent specificity and would allow to rule out infection reliably.

While this is a single-center study that uses a standardized diagnostic algorithm for PJI, it has several limitations. Firstly, patients were heterogeneous with regard to previous revision surgeries of a prosthesis, previous medical and surgical history, and microbiological findings. Secondly, several factors that might influence D-dimers were not investigated. While this might lessen the application of these findings to a general population, future studies should investigate typical D-dimer levels in patients with different anticoagulants, BMI, organisms, and based on age, sex, and ethnicity. The effect of anticoagulation could be of particular importance when the detection of an early infection is warranted as these patients usually still take postoperative prophylactic antithrombotic medication. Furthermore, while this study uses the MSIS criteria for the diagnosis of PJI, there are other criteria [37] that might be more accurate although there are no comparative studies to our knowledge.

In conclusion, we do not recommend routine use of D-dimers as a replacement of more established serum markers based on the findings from the present study. The diagnosis should be made using a set of synovial, serum, and microbiological findings. However, considering the limitations of the published studies, they might have a role as an add-on diagnostic tool that requires further investigation regarding an optimal cut-off value and potential adaptation for particular patient population or comorbidities.

## Figures and Tables

**Table 1:** Baseline demographics of the enrolled patients.

	PJI (*n* = 52)	Non-PJI (*n* = 67)	*p* Value
Mean age * (year)	70.5 (60.05–83.7)	68.0 (61.0–76.0)	0.499
Patients with Primary TJA	27 (51.9%)	29 (43.3%)	0.226
Patients with Revision TJA	25 (48.1%)	38 (56.7%)	0.362
Patients sex *			
Male	30 (57.7%)	39 (58.2%)	0.699
Female	22 (42.3%)	28 (41.8%)	
Mean BMI * (kg/m^2^)	30.9 (27.0 to 38.4)	28.4 (25.5 to 31.9)	0.008
Affected joint *			
Knee	30 (57.7%)	41 (61.2%)	0.711
Hip	22 (42.3)	26 (38.8%)	

* The median values are given as the number of cases and the percentage or IQR in parentheses. PJI, periprosthetic joint infection; TJA, total joint arthroplasty.

**Table 2:** Results for D-dimer, CRP, IL-6, and their combinations in diagnosing prosthetic joint infection (PJI).

Marker	Cut-off Used	Sensitivity (%)	Specificity (%)	PPV	NPV
D-dimer	2750 ng/mL	38	94	0.8333	0.6631
D-dimer	860 ng/mL	92	39	0.5393	0.8667
CRP	1.2 mg/dL	88	87	0.8364	0.9063
Il-6	10.0 pg/mL	76	92	0.8810	0.8261
CRP/Il-6	1.2 mg/dL/10.0 pg/dL	74	97	0.9474	0.8219
CRP/D-dimer	1.2 mg/dL/2750 ng/mL	35	96	0.8571	0.6531
Il-6/D-dimer	10.0 pg/dL/2750 ng/mL	33	97	0.8889	0.6451

CRP, C-reactive protein; PPV, positive predictive value; NPV, negative predictive value.

**Table 3:** Published results of serum D-dimer in diagnosing prosthetic joint infection (PJI) since its inclusion in the Musculoskeletal Infection Society (MSIS) criteria in 2018.

Author	Year	Sensitivity (%)	Specificity (%)	Used Cut-off Value [ng/mL]
Hu et al. [16]	2020	87.50	89.19	955
Qin et al. [17]	2020	92.72	74.63	1170
Li et al. [11]	2019	64.5	65.0	1250
Xu et al. [12]	2019	68.29	50.70	1020
Pannu et al. [18]	2020	95.9	32.3	850
